# Management of Diabetes Mellitus in Normal Renal Function, Renal Dysfunction and Renal Transplant Recipients, Focusing on Glucagon-Like Peptide-1 Agonist: A Review Based upon Current Evidence

**DOI:** 10.3390/ijms20133152

**Published:** 2019-06-28

**Authors:** Shang-Feng Tsai, Cheng-Hsu Chen

**Affiliations:** 1 Division of Nephrology, Department of Internal Medicine, Taichung Veterans General Hospital, Taichung 407, Taiwan; 2 Department of Life Science, Tunghai University, Taichung 407, Taiwan; 3 School of Medicine, National Yang-Ming University, Taipei 112, Taiwan

**Keywords:** diabetes mellitus, renal transplantation, new-onset diabetes after transplantation, glucagon-like peptide-1 agonist

## Abstract

Diabetes Mellitus (DM) is a leading cause of both Cardiovascular Disease (CVD) and End-stage Renal Disease (ESRD). After 2008, there has been much evidence presented, and recently the guidelines for sugar control have changed to focus on being more disease orientated. GLP-1 Receptor Agonists (GLP-1R) and sodium glucose cotransporter-2 inhibitors are suggested as the first line towards fighting all DM, CVD and ESRD. However, the benefits of GLP-1R in organ transplantation recipients remain very limited. No clinical trials have been designed for this particular population. GLP-1R, a gastrointestinal hormone of the incretin family, possesses antidiabetic, antihypertensive, anti-inflammatory, anti-apoptotic and immunomodulatory actions. There are few drug–drug interactions, with delayed gastric emptying being the major concern. The trough level of tacrolimus may not be significant but should still be closely monitored. There are some reasons which support GLP-1R in recipients seeking glycemic control. Post-transplant DM is due to an impaired β-cell function and glucose-induced glucagon suppression during hyperglycemia, which can be reversed by GLP-1R. GLP-1R infusion tends to relieve immunosuppressant related toxicity. Until now, in some cases, glycemic control and body weight reduction can be anticipated with GLP-1R. Additional renal benefits have also been reported. Side effects of hypoglycemia and gastrointestinal discomfort were rarely reported. In conclusion, GLP-1R could be implemented for recipients while closely monitoring their tacrolimus levels and any potential side effects. Any added benefits, in addition to sugar level control, still require more well-designed studies to prove their existence.

## 1. Introduction

Diabetes Mellitus (DM) is one of the leading causes of Atherosclerotic Cardiovascular Disease (ASCVD), Heart Failure (HF), and End-stage Renal Disease (ESRD), with the prevalence of DM still on the increase [1]. Control of blood glucose is the major way to avoid ACVD, HF and Diabetic Kidney Disease (DKD) related ESRD. Recently however, many studies have been published explaining the pleotropic effects of new diabetic medications, in addition to their ability to control sugar levels, including EMPA-REG [2], DECLARE-TIMI 58 [3], CANVAS [4], CREDENCE [5] and LEADER [6,7]. However, the above studies focused on DM populations with native kidneys. For new-onset diabetes after transplantation (NODAT), there have been no studies conducted with a large population. All concepts for controlling NODAT were based on the mechanisms, pharmacokinetics and pharmacodynamics of medications in native DM patients. This is even rarer in new medications for DM, such as glucagon-like peptide-1 receptor agonist (GLP-1R). First of all, we will review the complicated relationship between DM, DKD and CVD, where the importance of NODAT should be elucidated. Now, it is the new era for DM control because we have then chance with one medication to stop DM, DKD and CVD. More importantly, we will focus on GLP-1R in native DM patients and patients with NODAT.

## 2. Complicated Relationship between DM, DKD and CVD

The crosstalk between organs is very important for clinicians, particularly when treating Cardiorenal Syndrome (CRS) [8]. There are five types of CRS. As for Type 5 CRS, the primary events include septic shock, DM, metabolic syndrome and vasculitis. Chronic Kidney Disease (CKD) and CVD also share similar risk factors, such as DM, hyperlipidemia, aging, smoking and a positive family history of the disease [9]. DM is the major cause of ESRD and CVD. The outcome of patients with ESRD is the worst in those with DM related ESRD [10]. There are many similarities between CKD and CVD, in addition to having the similar risk factors mentioned above. For example, CKD and CVD are both chronic diseases with frequent acute episodes (acute kidney injury (AKI) and acute decompensated HF) [11,12]. Also, the 5-year survival rates of both ESRD [13] and end-stage HF [14,15] are approximately 50%. The mechanisms for CRS include overactivity of the sympathetic nervous system, natriuretic peptide system and Renin-angiotensin-aldosterone System (RAAS) [16]. Furthermore, CKD causes the medial calcification of vessels, whereas metabolic syndrome induces intimal thicknesses (atherosclerosis) [17]. Both CKD and CVD can lead to vascular stenosis, medium and intima, respectively [17]. Last but not least, the major cause of death for ESRD is CVD, from CKD (not yet dialysis) [10] to ESRD (under dialysis) [18], even after renal transplantation [19]. The outcome of DM related ESRD is poorest for patients with renal dysfunction. In short, the major problem for CKD and CVD patients is having DM. Therefore, treatment of DM is important to avoid CKD and CVD. In addition to sugar level control, there are still some pleotropic effects for anti-diabetic agents such as renal or cardiac protection, particularly GLP-1R and a Sodium Glucose cotransporter-2 inhibitor (SGLT2i). Therefore, early usage of the above medications has been suggested in the clinical guidelines [20,21].

## 3. New Ear for DM Control 

After the cardiovascular concerns surrounding Muraglitazar [22] and Rosiglitazone [23], the U.S. Food and Drug Administration (FDA) regulated that any new medication for DM should focus on CV Outcome Trials (CVOTs) in order to rule out unacceptable CV risk (non-inferiority study). Therefore, after 2008, any new medications for DM all had to have similar study designs and outcome analysis, such as Major Adverse Cardiac Event (MACE). After the 2008 FDA guidelines were written, nearly all Dipeptidyl Peptidase-4 Inhibitors (DPP4i (SAVOR-TIMI 53 [24], EXAMINE [25], TECOS [26], and CARMELINA [27]), SGLT2i (EMPA-REG [2], CANVAS [4], DECLARE-TIMI58 [3], and CREDENCE [5]) and GLP-1R (LEADER [6], ELIXA [28], SUSTAIN-6 [29], and EXSCEL [30]) have had similar study designs of CVOTs [31]. Overall, more than 190,000 participants with DM were enrolled in CVOTs after 2008. Initially, CVOTs were designed as a non-inferiority study as primary outcome. However, some studies (LEADER [6], SUSTAIN-6, EMPA-REG [2], CANVAS [4], and DECLARE-TIMI-58 [3]) had additional CV or renal benefits (superior benefit), in addition to sugar level control. Therefore, the US based American Diabetes Association (ADA) [20], American Association of Clinical Endocrinologists and American College of Endocrinology [21] all made major shifts in the algorithm of sugar control in Type 2 DM. After the first line of treatment (metformin), it should be disease-orientated [20,21]. If diabetic patients have ASCVD, HF or CKD, clinicians should first prescribe GLP-1R or SGLT2i. It is a new era for diabetic care due to the additional CV and renal benefits independent from sugar level control. As for GLP-1R, in addition to sugar control, the CV protection maybe through blood pressure control, body weight reduction and diabetic dyslipidemia [32]. It can regulate cholesterol and triglycerides by numerous ways. Liraglutide was also reported to decrease lipid profile and improve leptin and adiponectin levels [33]. In current ear for the treatment of DM, we have a chance to control DM, ASCVD, HF and DKD with only one medication. However, all of the above studies were conducted in DM patients with native kidneys. None of them were for NODAT or DM with allograft kidneys.

## 4. DM Control in DM-CKD

Sugar level control in renal dysfunction is further complicated, including those patients with native kidneys and allography kidneys. Firstly, in early CKD, less insulin secretion and more insulin resistance requires patients to need a higher insulin dose or more medications for sugar level control [34]. However, as renal function further declined, less gluconeogenesis was noticed [35], and a longer half-life of insulin both made for a lesser insulin requirement in advanced CKD patients. Eventually, when the Glomerular Filtrate Rate (GFR) was less than 10 mL/min, patients only required 50% of the insulin dosage [36]. Secondly, less strict sugar level control was needed for advanced CKD patients [37]. The phenomenon of the J curve for glycated hemoglobin (HbA1c) was also noticed in CKD patients [38]. Finally, HbA1 will be underestimated in advanced CKD patients [39]. The above condition will require more attention being paid to DM-CKD patients in both native kidneys and allography kidneys.

In advanced CKD patients, only certain medications can be used [40], including all DPP4is, Repaglinide, Glipizide, Gliclazide, Pioglitazone, GLP-1R (Liraglutide and Dulaglutide) and insulin. The FDA approved their usage based on pharmacokinetic studies at the least or Phase 3 randomized clinical trials. We will focus on GLP-1R in the following discussion.

## 5. New-Onset Diabetes after Transplantation (NODAT)

**Figure 1:**
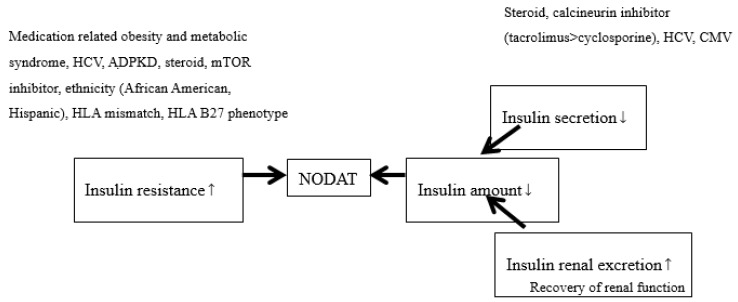
The pathogenesis of new-onset diabetes after transplantation (NODAT). Smaller arrows indicate increase (upward) and decrease (downward). Larger arrows indicate the cause.

After the improvements were seen in both patient and graft survival after transplantation, non-immunologic outcomes became important, including NODAT (formerly called post-transplant DM). NODAT causes a higher rate of CVD and infection and is a major cause of morbidity and mortality. According to a consensus regarding NODAT in 2003, HbA1c was not recommended within three months after transplantation [41]. The primary reason is that during that period, renal function was still under recovery, and HbA1c may underestimate sugar control. The incidence of NODAT varied (10% to 74%) [42] according to the different times of diagnosis after transplantation, different definitions, different immunosuppressants and different patient demographics. It increased as time went on [43]: 9% after 3 months, 16% after 12 months and 36% after 36 months. Nearly all treatments for NODAT were based on studies of non-transplanted patients. Reasonably, the sugar control, renal benefits and CV benefits for sugar control in non-transplanted patients can be extended to NODAT. An all new algorithm regarding sugar control in the ADA guidelines [20] can be used in NODAT; however, more well-designed studies are still required in this special population to obtain more evidence. In other words, any additional CV and renal benefits from GLP-1R and SGLT2i for NODAT also require more studies in the future. The pathogenesis of NODAT is summarized in Figure 1.

## 6. GLP-1R for Sugar Control in DM-CKD

GLP-1R, a gastrointestinal hormone of the incretin family, offers antidiabetic, antihypertensive, anti-inflammatory, anti-apoptotic and immunomodulatory actions [44]. Exenatide and Exenatide extended release have been approved by the US FDA as adjunctive therapy for patients with Type 2 DM, but should be avoided in patients with a creatinine clearance <30 mL/min because both are eliminated through the kidneys [45]. That would cause the accumulation of exenatide, followed by more nausea and vomiting related to pre-renal AKI. The AKI will lead to more blood concentration of Exenatide. Furthermore, no data is yet available for use of Exenatide in kidney transplant recipients, particularly during the period of fluctuating renal function. Until now, more than 78 cases with AKI have been reported [46]. Therefore, in our opinion we do not recommend Exenatide for patients, particularly those experiencing renal function fluctuation, as it will cause non stationary plasma concentration. However, both Liraglutide and Dulaglutide can be used because they are only degraded by endogenous proteolysis without specific organs. Accordingly, they may also be used for advanced CKD, and even for ESRD patients, when implemented with caution.

In the LIRA-RENAL trial for type 2 DM [47], no patients were enrolled if their GFR was less than 30 mL/min.1.732 m^2^. In the LEADER trial [6], only 2.5% patients had a GFR< 30 mL/min.1.732 m^2^. In a recent study [48], plasma Liraglutide concentrations increased during treatment in patients with Type 2 DM and ESRD, which caused an increase in nausea and possible renal injury. In clinical practice, reduced treatment doses and a prolonged titration period still remains necessary. As for Dulaglutide, there has been a limited experience in patients with Stages 4–5 CKD in 2016 [49]. However, in AWARD-7 for type 2 DM [50,51], 70% of patients were Stage 3-CKD and 30% were Stage 4-CKD. Dulaglutide produced glycemic control similar to that achieved with insulin glargine, with a reduced decline in eGFR. It was also safe to use in order to achieve glycemic control in this population, while providing a lesser observed decline in eGFR.

## 7. GLP-1R for Recipients

Until now, there had been no trials for GLP-1R with regards to glycemic control for recipients. Evidence for this issue has been based upon only certain case series. Many parts’ concerns still need to be clarified before using GLP-1R in transplantation recipients. Firstly, there was no hepatic metabolism and additionally, much less cytochrome P450 3A4 enzyme related drug–drug interaction (DDI). These do not engage in cytochrome- or transporter-mediated DDIs [52]. This was particularly good for both tacrolimus, and cyclosporine.

However, one major concern is that GLP-1R will slow gastric emptying (particularly during the first one or two doses of Lxenatide and Lixisenatide [53]), which will affect immunosuppressant absorption. Tacrolimus in particular has a narrow therapeutic index. A delayed drug concentration may be experienced due to delayed gastric emptying, but the drug exposure may not affected [54,55]. Pinelli Pinelli et al. in 2013 [56], reported on 5 cases with NODAT which were exposed to concomitant Liraglutide and tacrolimus. Tacrolimus AUC_0–12h_ appeared reduced after Liraglutide had been administered, although the trough concentrations were unchanged. Also, no acute rejection was noticed. Similarly, in another study in 2018 [57], Chen CH et al. demonstrated a study for NODAT or preexisting type 2 DM in recipients that a steady state tacrolimus level, although 3 of 5 recipients had to reduce their dose of tacrolimus. Therefore, even though there was limited clinical significance for this GLP-1R related delayed gastric emptying, we still strongly suggested the need to closely monitor the trough level of tracrolimus, if co-administered with GLP-1R.

There still remain some reasons for using GLP-1R in glycemic control of recipients. Firstly, NODAT was due to an impaired β-cell function and glucose-induced glucagon suppression during hyperglycemia [58,59]. In a study in 2016 [58], after a 3-h intravenous infusion of GLP-1R, GLP-1R infusion tended to improve insulin and glucagon effects in recipients with NODAT. Secondly, a study of insulinoma cells in mice showed that pancreatic β-cells expressing GLP-1 are resistant to the toxic effects on islet cell of immunosuppressive drugs [60]. The effect of dexamethasone in inducing cell death in insulin-secreting cells can be reversed through the use of exendin-4 [61]. In an animal study in 2015 [62], DPP4i played an important role in the renoprotection against tacrolimus-induced nephrotoxicity, via its antioxidative and antiapoptotic effects and preservation of the GLP-1 system. This effect can also be observed in a human study for eight healthy men [63] which showed that Exenatide prevented both prednisolone-induced glucose intolerance and islet-cell dysfunction. GLP-1R may target the pathogenesis of NODAT. In a retrospective and observational study performed in 2014 [64], 20 post-transplant recipients (7 kidneys) with preexisting type 2 DM or NODAT were given GLP-1R (Liraglutide or Exenatide). Here, HbA1c and weight loss (19 of 20 patients) were experienced. The maximum weight loss was 33.4 lbs. The Serum creatinine (Scr) and tacrolimus levels were similar. Only two patients underwent dose reduction due to negative side effects, while no events of pancreatitis were reported. The baseline renal function was good, 1.3 ± 0.5 mg/dL. In another study by Chen et al. in 2018 [57], 7 cases with with preexisting type 2 DM or NODAT were reported using Liraglutide, which was safe and effective for glycemic control (*p* = 0.043), with some intolerance (2 of 7 patients). There was also improved graft renal function, and a significantly improved eGFR, from 67.7 ± 18.7 to 76.5 ± 18.7 mg/dL (*p* = 0.024). No hypoglycemia was noticed. Until now, the largest case series (63 recipients who had received Dulaglutide regardless of the time of onset of diabetes with respect to a transplant) to be reported was published in 2018 [65]. The baseline renal function was good, 1.55 mg/dL of Scr. The body weight was reduced, and insulin reduction before and after Dulaglutide treatment was also significant (*p* < 0.0002). Gastrointestinal manifestations were rare. In summary, glycemic control and body weight reduction can be anticipated in GLP-1R recipients. All human studies regarding recipients using GLP-1R is summarized in Table 1.

SGLT2i had been reported to exhibit renal protection in patients with preexisting type 2 DM in major studies including EMPA-REG [2], DECLARE-TIMI 58 [3], CANVAS [4], and CREDENCE [5]. Some studies [66,67] mentioned that combing SGLT2i and GLP-1R as a treatment for preexisting type 2 DM yielded better sugar and blood pressure control, increased body weight reduction and reduced CV risk synergistically. However, this result still lacks large prospective study. Therefore, since the evidence of GLP-1R in renal recipients is rare, studies with the combination of SGLT2i and GLP-1R is even rarer.

## 8. Conclusions

DM is the leading cause of ASCVD, HF and ESRD. The association amongst DM, HF and CKD is very close and complicated. Currently, GLP-1R and SGLT2i are suggested as the first options when attempting to stop all three situations. However, studies on the use of GLP-1R in recipients are still quite limited. GLP-1R may target the pathogenesis of NODAT and can be considered for glycemic control in recipients. However, delayed gastric emptying may influence the concentration of immunosuppressants and should be closely monitored.

## Figures and Tables

**Table 1:** All published studies regarding recipients using GLP-1R.

Study	Case Number	Medication	Renal Function	Sugar Control	Body Weight Reduction	Additional Benefits	Side Effects
van Raalte et al. [63], 2011	8	Exenatide	n/a	Better	n/a	n/a	n/a
Pinelli NR et at. [56], 2013	5	Liruglutide	eGFR = 70–116 mL/min.1.732 m^2^	n/a	n/a	n/a	No hypoglycemia
Krisl et al. [64], 2014	20	Liraglutide, exenatide	Scr = 1.3 ± 0.5 mg/dL	n/a	maximum weight loss was 33.4 lbs.	ns	no pancreatitis, no hypoglycemia
Halden et al. [58], 2016	12	Lyophilized GLP-1 (7–36) amide	eGFR = 69 ± 12 mL/min.1.732 m^2^	*p* ≤ 0.001	n/a	n/a	No hy poglycemia
Chen et al. [57], 2018	7	Liraglutide	eGFR = 67.7 ± 18.7 mL/min.1.732 m^2^	*p* = 0.017	*p* = 0.032	Better eGFR	No hypoglycemia; 28.6% discontinue
Priyamvada et al. [65], 2018	63	Dulaglutide	Scr = 1.55 mg/dL	*p* < 0.0002	*p* < 0.034	n/a	6.3% non-severe hypoglycemia; 1.5–3% GI discomfort

n/a: not available.
